# Accurate Prediction of Three-Dimensional Humanoid Avatars for Anthropometric Modeling

**DOI:** 10.21203/rs.3.rs-4565498/v1

**Published:** 2024-07-13

**Authors:** Steven Heymsfield, Cassidy McCarthy, Michael Wong, Jasmine Brown, Sophia Ramirez, Shengping Yang, Jonathan Bennett, John Shepherd

**Affiliations:** Pennington Biomedical Research Center; Pennington Biomedical; University of Hawaii Cancer Center; Pennington Biomedical Research Center

**Keywords:** Body Composition, Nutritional Assessment, Obesity, Energy Balance, 3-Dimensional Optical

## Abstract

**Objective:**

To evaluate the hypothesis that anthropometric dimensions derived from a person’s manifold-regression predicted three-dimensional (3D) humanoid avatar are accurate when compared to their actual circumference, volume, and surface area measurements acquired with a ground-truth 3D optical imaging method. Avatars predicted using this approach, if accurate with respect to anthropometric dimensions, can serve multiple purposes including patient metabolic disease risk stratification in clinical settings.

**Methods:**

Manifold regression 3D avatar prediction equations were developed on a sample of 570 adults who completed 3D optical scans, dual-energy X-ray absorptiometry (DXA), and bioimpedance analysis (BIA) evaluations. A new prospective sample of 84 adults had ground-truth measurements of 6 body circumferences, 7 volumes, and 7 surface areas with a 20-camera 3D reference scanner. 3D humanoid avatars were generated on these participants with manifold regression including age, weight, height, DXA %fat, and BIA impedances as potential predictor variables. Ground-truth and predicted avatar anthropometric dimensions were quantified with the same software.

**Results:**

Following exploratory studies, one manifold prediction model was moved forward for presentation that included age, weight, height, and %fat as covariates. Predicted and ground-truth avatars had similar visual appearances; correlations between predicted and ground-truth anthropometric estimates were all high (R^2^s, 0.75–0.99; all p < 0.001) with non-significant mean differences except for arm circumferences (%D ~ 5%; p < 0.05). Concordance correlation coefficients ranged from 0.80–0.99 and small but significant bias (p < 0.05 – 0.01) was present with Bland-Altman plots in 13 of 20 total anthropometric measurements. The mean waist to hip circumference ratio predicted by manifold regression was non-significantly different from ground-truth scanner measurements.

**Conclusions:**

3D avatars predicted from demographic, physical, and other accessible characteristics can produce body representations with accurate anthropometric dimensions without a 3D scanner. Combining manifold regression algorithms into established body composition methods such as DXA, BIA, and other accessible methods provides new research and clinical opportunities.

## INTRODUCTION

The recent introduction of low-cost three-dimensional (3D) optical imaging systems is revolutionizing anthropometric assessment for children and adults [1–4]. These digital systems, some stationary [5] and others housed in smartphones [6], can capture a person’s whole-body surface data to create a 3D humanoid avatar and estimate anthropometric measures across the whole body using the acquired information [7]. As a result of these technological advances, large 3D avatar databases are accumulating as scanning technology becomes increasingly available in research and clinical settings. One application of these archived humanoid avatars is to serve as a reference sample for developing manifold regression equations that can be used to predict a person’s physical representation in 3D from their demographic (e.g., sex, age, etc.), physical (e.g., weight, height, etc.), and other accessible characteristics (e.g., %fat, segmental impedance, body density, etc.) without requiring 3D scanning equipment [8, 9]. A question that arises is how accurate are the predicted 3D renderings of the human body with respect to commonly evaluated anthropometric dimensions including body circumferences, volumes, and surface areas? Accurately predicting a person or group’s anthropometric features acquired from their 3D avatar would have several potential research and clinical applications. Accordingly, the aim of the present study was to evaluate the hypothesis that anthropometric dimensions derived from manifold-regression predicted 3D humanoid avatars are accurate with respect to measures collected with a ground-truth imaging method.

## METHODS

### Study Design

**Figure 1:**
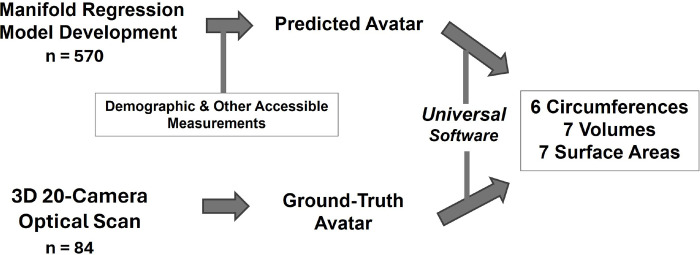
Experimental study plan. The first study phase involved development of manifold regression models and the second phase involved comparisons of predicted and ground-truth avatar body circumferences, volumes, and surface areas.

The study design is summarized in Fig. 1. The first study phase involved development of manifold regression prediction models on a sample of healthy adults. Additional information is provided in Methods on development of the manifold regression models. The second prospective phase then followed with comparison of predicted avatar anthropometric dimensions (6 circumferences, 7 volumes, and 7 surface areas) to corresponding ground-truth estimates in a new sample of healthy adults. Ground-truth anthropometric measurements were acquired with a 20-camera 3D optical scanner (SS20, Size Stream, Cary, NC). Predicted avatars were developed by manifold regression using several different exploratory combinations of demographic, physical, and other accessible characteristics as described in the Methods section. Accessible characteristics in the current study were acquired with dual-energy X-ray absorptiometry (DXA, QDR Discovery, Hologic, Marlborough, Massachusetts) and bioimpedance analysis (BIA, InBody S10, Seoul, South Korea). The predicted and actual 3D avatars were analyzed using the same Universal Software [10, 11] developed to identify standard anatomic landmarks.

### Participants

In the first phase of the study, manifold regression model development, participants were evaluated as part of the cross-sectional Shape Up! Adults study (NIH R01 DK109008). The Shape Up! Adults study was designed to investigate associations between body shape and composition with multiple health markers [4, 9]. In the second phase of the study, avatar anthropometric evaluation, participants were a new prospectively evaluated sample of healthy adults at or over the age of 18 years who completed the protocol measurements on the same day. These participants were recruited from the local community through web postings and print media. All participants enrolled in the study self-reported their race/ethnicity. The parent study for this project was approved by the Pennington Biomedical Research Center and University of Hawaii Cancer Center Institutional Review Boards and is posted on ClinicalTrials.gov (ID NCT03637855). The second phase of the current study was approved by the Pennington Biomedical Research Center Institutional Review Board (IRB# PBRC 2022–002). Baseline evaluations included health screening and measurement of body weight and height.

### Manifold Regression Model Development

#### Statistical Shape Model

After 3D optical data acquisition, each scan was registered to a 60,001-vertex template using the methods of Allen et al. [12]. This standardization allowed direct anatomical body shape comparisons across the sample. First, seventy-five fiducial points defined in the Civilian American and European Surface Anthropometry Resource Project [13] were manually placed on the raw meshes by trained and validated personnel using Meshlab 1.3.2 (Consiglio Nazionale delle Ricerche, Rome, Italy). Using the software Ganger, developed by Allen et al. [12], the template’s markers were transformed to each target mesh’s markers. The vertices of the template warps to fit the shape of each participant’s mesh [14]. Next, a principal component (PC) transformation of the meshes was performed to create sex-specific statistical shape models. These models described 99% of the body shape variance using fewer than 15 PCs [15].

#### Manifold Matrix

Manifold regression analysis was performed following the creation of the shape models. The manifold equation is *M = P × F*^*+*^, where Mis the manifold, *P* is the matrix of all PCs for all participants in the shape model, *F* is the matrix of all feature parameters (e.g., height and weight) for all participants, and ^*+*^ symbolizes the pseudoinverse. Once *M was* calculated, another matrix was created, *W* which contained the target features from a person’s feature parameters (e.g., height = 150 cm and weight = 60 kg). Matrix, *M,* was then multiplied to matrix *W*, creating a new PC matrix where the target features of *W* have modified *M.* The new PC matrix was then transformed back into Cartesian space from the PC space to generate the manifold images [16, 17].

#### Avatar Features

**Figure 2:**
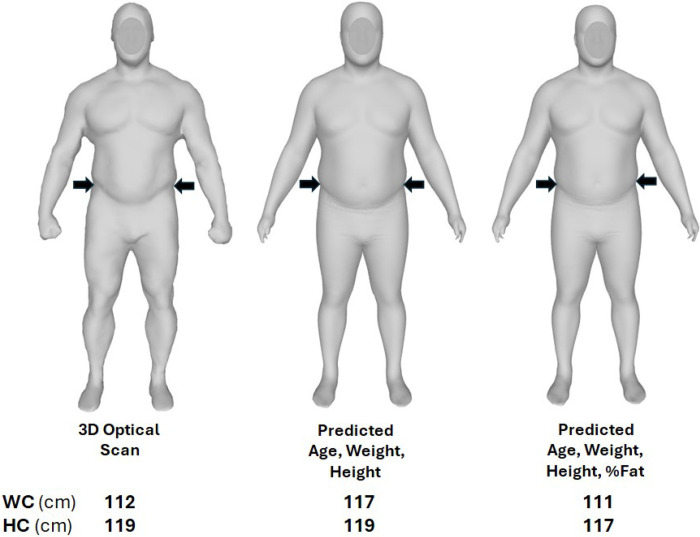
Example of avatars generated using two different manifold regression equation inputs and an actual 3D optical scan. The evaluated man was a body builder (left panel) with a waist circumference of 86.9 cm (designated by arrows) and hip circumference of 101.4 cm; his body fat was 18% and body mass index 35.5 kg/m^2^. Manifold-predicted waist and hip circumferences (middle panel) were 89.2 cm and 101.9 cm, respectively, with age, weight, and height as covariates. Manifold-predicted waist and hip circumferences (right panel) were 86.7 cm and 101.1 cm, respectively, with %fat added to the age, weight, and height covariates. Adding %fat to the model including age, weight, and height as covariates brought visual appearance closer to actual appearance and improved waist circumference prediction by ~2 cm.

The manifold regression models can predict 3D humanoid avatars using demographic covariates such as age and physical characteristics including weight and height. Additional characteristics can be included in the equations such as %fat and impedance values from BIA. Adding more covariates usually refines predictions, especially in samples that have highly varied body shapes. In the current study, we found in exploratory evaluations that the simplest model giving good anthropometric predictions relative to ground-truth included age, weight, height, and %fat (DXA) as covariates. Since the shape models were sex-specific, sex was not used as a covariate. This four-variable model was created by modifying F in the manifold equation. An example of the difference in predicted avatars between a model with age, weight, and height and a model that additionally included %fat is shown in Fig. 2 for a young muscular adult male. The three-variable model did not distinguish people in the current study who were muscular from their counterparts with greater relative adiposity as was observed in the participant presented in the figure. Manifold regression analysis was performed in R version 4.2.1 (https://stat.ethz.ch/pipermail/r-announce/2020/000658.html; R Core Team, 2020).

#### Universal Software

Anthropometric body dimensions were evaluated in the predicted and ground-truth avatars with Universal Software. This software operates on Matlab (Mathworks, Natick, MA) [10, 18] and runs four sequences including pre-processing, landmark detection, body partitioning, and surface area calculation. Initial scan processing repairs gaps or imperfections in the 3D mesh. Major anatomic landmarks are next detected [10] at the crotch, right/left armpits, shoulders, hips, and toes. The software then partitions body mass into six regions including head-neck, trunk, right/left arm, and right/left leg followed by calculation of body lengths, 6 circumferences (waist, hip, right/left mid-upper arm, right/left thigh) and 7 regional/total volumes (head/neck, torso, right/left arms and legs, whole-body), and the same 7 regional/total surface areas. The circumference sites are shown in **Supplementary Information I** and **Figure S1**.

#### Measurements

The SS20 3D optical reference system includes twenty structured light infrared depth sensors mounted on four vertical columns. Participants stood in the A-pose at the center of the columns and data was acquired during a 4-second scan. The acquired avatars were analyzed using Universal Software.

The QDR Discovery DXA was operated with software version V8.26a:3.19 and calibrated at regular intervals according to manufacturer specifications. The National Health and Nutrition Examination Survey scanner option was turned off. Two components were evaluated, total body fat and fat-free mass; percentage (%) fat mass was derived as (fat mass/body mass) × 100.

The InBody S10 BIA system used in exploratory studies has touch-type electrodes that are attached between the heel and ankle bone of the participants’ feet and on the middle finger and thumb of each hand. Impedance of the right arm, left arm, right leg, left leg, and trunk were measured at frequencies of 1, 5, 50, 250, 500, and 1000 kHz. Model exploratory evaluations were completed with data acquired at the commonly used frequency of 50 kHz.

#### Statistical Methods

Avatars created using manifold regression (predicted) were compared with the actual (ground truth) participant avatars for selected circumferences, volumes, and surface areas using linear regression analysis (R^2^) and with means (± SD), root-mean square errors (RMSEs), mean absolute errors (MAEs, X ± SE), concordance correlation coefficients (CCCs), and Bland-Altman analyses [19]. The predicted and ground-truth avatar comparisons are presented separately for the circumferences and combined for the volumes and surface area evaluations.

## RESULTS

### Sample Characteristics

The sample used to develop the manifold regression model consisted of 570 adults, including 258 males and 312 females (**Table S1**). The sample in the second study phase included 84 adults, 35 males and 49 females, with a mean age of 45 years (**Table S2**). Males had a larger body mass index than females (~ 30 vs. 25 kg/m^2^) whereas females had higher percent body fat (~ 35 vs 28%). There were 70 White, 8 Black, and 6 Asian participants.

### Circumference Evaluations

**Figure 3:**
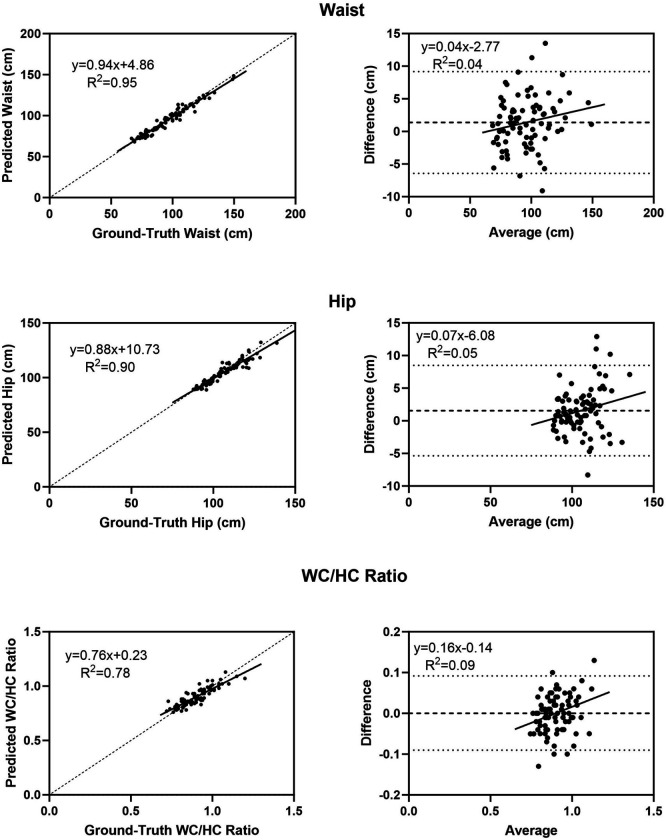
Predicted versus ground-truth body circumferences for the waist (WC), hip (HC), and waist to hip ratio (left) and corresponding Bland-Altman plots (right). Regression equations and R^2^ values are shown in each panel of the figures and p-values are provided in Table 1. The regression line in the lefthand panels is in bold and the line of identity is dashed. The mean difference (bold) and 95% confidence intervals are shown in the righthand panels.

The results of predicted versus ground-truth avatar circumferences are shown in Table 1 as the mean ± SDs, MAEs, RMSEs, CCCs, and Bland-Altman analyses. The correlations and concordance between predicted and ground-truth circumference estimates were all high with R^2^s ranging from 0.78 to 0.95 (all p < 0.001) and CCCs ranging from 0.80 to 0.97. Lower correlations tended to be present in both arms (R^2^, ~ 0.78) that also showed small significant (~ 5%, p < 0.05) mean differences between predicted and ground-truth circumferences. There were no other significant predicted-ground-truth mean circumference differences, with small MAEs (2.2–3.3 cm) and RMSEs (2.9–4.2 cm). The correlation between the predicted and measured waist to hip circumference ratio had an R^2^ and CCC of 0.77 and 0.87, respectively; significant bias (p < 0.01) was present with a mean bias of 0.001 cm. Figure 3 provides plots of predicted versus ground-truth waist and hip circumferences and the waist to hip circumference ratio. Significant (p < 0.05 – 0.01) bias observed with the Bland-Altman plots was present for the hip (Fig. 3), arm, and thigh circumferences with respective mean biases of 0.2–2.0 cm. Examples of generated images with waist and hip circumference measurements and the waist to hip ratio are shown for a representative male and female in **Figure S2**. Predicted and ground-truth circumferences observed in the male are in good agreement while the predicted female avatar visually appears leaner than the ground-truth avatar and this leaner appearance is reflected by smaller waist (6–7%) and hip (2–3%) circumferences and a smaller waist to hip circumference ratio (3–4%).

### Volume and Surface Area Evaluations

**Figure 4:**
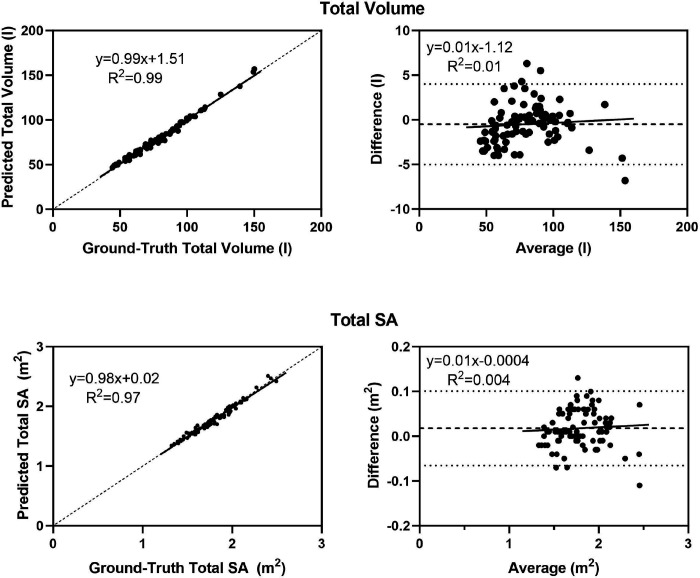
Predicted versus ground-truth total body volume and surface area (SA) and corresponding Bland-Altman plots (right). Regression equations and R^2^ values are shown in each panel of the figures and p-values are provided in Table 2. The regression line in the lefthand panels is in bold and the line of identity is dashed. The mean difference (bold) and 95% confidence intervals are shown in the righthand panels.

The results of predicted versus ground-truth avatar volumes and surface areas are shown in Table 2. Ten measurements of right leg volume on the SS20 scanner were technically inadequate and the sample in this cell is reduced accordingly. The correlations and concordance between predicted and ground-truth regional and total volume estimates were all high with R^2^s ranging from 0.77 to 0.99 (all p < 0.001) and CCCs ranging from 0.87 to 0.99. There were no significant predicted-ground-truth mean volume differences with small MAEs (0.01–0.1 l) and RMSEs ranging from 0.01 to 0.06 l. Significant (p < 0.05 – 0.01) bias observed with the Bland-Altman plots was present for the head, arm, trunk, and leg with respective mean differences of −0.2–0.21. The correlation between predicted and ground-truth total volume had an R^2^ (Fig. 4) of 0.99 also a CCC of 0.99; non-significant bias was present with a mean difference of −0.5 l.

The correlations and concordance between predicted and ground-truth regional and surface area estimates were all high with R^2^s ranging from 0.74 to 0.97 (all p < 0.001) and CCCs ranging from 0.87 to 0. 99. There were no significant predicted-ground-truth mean surface area differences with small MAEs (0.01–0.04 m^2^) and RMSEs ranging from 0.01 to 0.06 m^2^. Significant (p < 0.05) bias observed with the Bland-Altman plots was present for the head, leg, and trunk with respective mean differences of 0.003–0.05 m^2^. The correlation between predicted and measured total surface area had an R^2^ (Fig. 4) and CCC of 0.97 and 0.99, respectively; non-significant bias was present with a mean difference of 0.02 m^2^.

### Composite Summary

Overall, differences in the mean predicted and ground-truth circumference, volume, and surface area evaluations were all non-significant, except for the two arm circumferences (D, ~ 5%). All of the other measures of agreement were good-to excellent, although small significant bias was present across all three types of anthropometric measurements, primarily those of the arms and legs.

## DISCUSSION

Advances in 3D optical imaging are providing an unprecedented opportunity to amass large databases of humanoid avatars that can be used as reference samples for developing manifold regression prediction models such as those reported in the current study. While the produced images in earlier studies appeared visually accurate [8, 9], a critical question remained: are predicted avatars also accurate with respect to actual physical dimensions? The current study was designed to examine this question by comparing circumferences, volumes, and surface areas on humanoid avatars generated by manifold regression to corresponding measurements made on avatars acquired in healthy adults with a 20-camera 3D optical scanner; identical software was used to process predicted and ground-truth avatars. Our findings answered the question affirmatively: group mean values for 6 circumferences, 7 volumes, and 7 surface areas observed in 84 adults did not differ significantly from those acquired with the optical scanner except for arm circumferences (D, ~ 5%). While measures of agreement such as R^2^s, RMSEs, and CCCs were all strong for predicted versus actual avatars, there was significant bias detected on several of the digitally estimated anthropometric measurements. These small bias effects can potentially be reduced or even eliminated in future studies by expanding the manifold regression sample and/or adding more or different accessible features to the developed prediction equations. Accessible features that could serve as regression model covariates, other than DXA, include multiple different or combined resistance, reactance, and phase angle whole-body and regional values at a range of frequencies acquired with BIA, %fat as measured with BIA, and body density and %fat as quantified with air-displacement plethysmography (ADP). Evaluating the many potential manifold regression model covariates and interactions was beyond the scope of the present study but would be an appropriate follow-up investigation aimed at furthering the accuracy of predicted avatar anthropometric dimensions.

### Potential Applications

The current study was prompted by earlier reports employing visual aspects of digital human avatars [8, 9, 20–23]. Our findings show that generated digital humanoid avatars can also have accurate physical dimensions that can prove useful in research and clinical settings. One application is with dynamic energy balance models that include predictions of long-term weight and %fat changes with lifestyle and pharmacologic treatments [24, 25]. These dynamic models can be supplemented with visually accurate avatars that could additionally provide information on baseline and follow-up body dimensions such as the waist to hip circumference ratio, a marker of metabolic and disease risks [26]. Three-dimensional models of human thermoregulation are now also being introduced for physiological, medical, and public health applications for which accurate anthropometric features as shown in the current study are important for accurate predictions [27]. Developed avatars in exploratory modeling studies could be further processed to show before-after pseudo-DXA [28] and whole-body skeletal images [29] that can have research and educational value. These pseudo-images mimic their actual counterparts and can be generated from the 3D avatar digital outputs.

Another group of applications prevails in the areas of obesity and eating disorders were visualizations of humanoid avatars are now being included in patient evaluation and management studies. Horne et al. [22] found that seeing a “future self” in the form of a personalized avatar reinforced motivation to modify behavior and promote engagement in a weight loss program. Three-dimensional avatars are also being used to visually map body image perceptions as an objective means of revealing anorexia nervosa illness severity [20, 21,30]. Manifold regression models such as those evaluated in the current report have the potential to improve the visual and anthropometric accuracy (e.g., as for the participant presented in Fig. 2) of the avatars generated in these studies.

Lastly, outside of research laboratories, can our avatar approach when combined with widely available non-X-ray body composition methods such as BIA and ADP give sufficiently accurate anthropometric estimates (e.g., waist circumference) to improve the clinical diagnosis and monitoring of patients with overweight and obesity? Neither of these methods provide patient visualizations, circumferences, or surface areas and manifold regression predictions would thus be complementary to these respective device outputs. Avatars with accurate anthropometric dimensions could also be generated on large population samples such as the U.S. National Health and Nutrition Examination Survey that could yield a wealth of information useful in multiple contexts. An essential step in this process, as noted earlier, would be to further improve manifold regression models with larger and more diverse development samples. An important question that also needs to be explored is if combinations of data from methods such as BIA and ADP with those provided by a predicted or actual 3D avatar can improve estimates of body composition and link these evaluations closer to functional and clinical outcomes [31].

### Study Limitations

Several limitations of the current study should be noted. First, our sample for developing manifold regression models was relatively small and limited to healthy adults. Anthropometric predictions would likely be less accurate in samples including older frail adults, people with dysmorphic body shapes such as scoliosis or lipedema, and persons with severe obesity or who are pregnant are some examples. Furthermore, the predicted avatars only provide an estimate based on a global average. For those with unique body shape characteristics, such as a panniculus present in the male in **Figure S2**, the prediction model could create an inaccurate 3D avatar as the majority of participants in the modeling population might not exhibit this characteristic. Future studies similar to the current investigation could also explore avatar anthropometric predictions in children and adolescents.

### Conclusions

The current study confirms that the anthropometric dimensions of 3D humanoid avatars derived from manifold-regression are accurate on average, although with some small bias for some measurements, with respect to a ground-truth optical imaging method. Future studies are needed to refine our proof-of-concept findings and models with larger participant samples and additional model covariates. Combining manifold regression algorithms with established body composition modalities such as DXA, BIA, and ADP may provide new insights and clinical opportunities.

## Figures and Tables

**Table 1:** Results of digital circumference evaluations in the prospective sample.

Circumference	Ground-Truth (mean ± SD, cm)	Predicted (mean ± SD, cm)	MAE ± SD (cm)	RMSE (cm)	R^2^***	CCC	Bias (mean ± SD, cm)
**Waist**	96.4 ± 18.3	95.1 ± 17.5	3.3 ± 2.6	4.2	0.95	0.97	1.4 ± 4.0
**Hip**	106.1 ± 11.2	104.6 ± 10.4	2.8 ± 2.6	3.8	0.90	0.97	1.6 ± 3.5*
**R Thigh**	57.1 ± 7.8	56.8 ± 6.3	2.2 ± 1.8	2.8	0.88	0.92	0.3 ± 2.8**
**L Thigh**	57.0 ± 7.8	56.7 ± 6.2	2.2 ± 2.0	2.9	0.88	0.91	0.2 ± 3.0**
**R Arm**	34.4 ± 6.1	32.4 ± 5.1^1^	2.3 ± 2.6	3.5	0.78	0.82	1.9 ± 2.9**
**L Arm**	35.0 ± 6.1	32.9 ± 4.7^1^	2.4 ± 2.5	3.5	0.79	0.80	2.0 ± 2.9**
**WC/HC**	0.90 ± 0.10	0.90 ± 0.08	0.04 ± 0.03	0.05	0.77	0.87	0.001 ± 0.05**

1 p < 0.05 for comparison of mean circumfernces

* p < 0.05 and

** p < 0.01 for Bland-Altman slope

*** all p < 0.001.

Abbreviations: CCC, concordance correlation coefficient; GT, ground-truth; HC, hip circumference; L, left; MAE, meanabsolute error; Pred, predicted; R, right; RMSE, root-measn square error; WC, waist circumference.

**Table 2:** Results of regional and whole-body digital volume (Vol) and surface area (SA) evaluations.

Volume	Ground-Truth (mean ± SD, L/m^2^)	Predicted (mean ± SD, L/m^2^)	MAE ± SD OMm^2^)	RMSE (L)/(m^2^)	R^2^***	CCC	Bias (mean ± SD, L/m^2^)

**Total**	**79.2 ± 23.0**	**79.7 ± 22.8**	**1.8 ± 1.5**	**2.3**	**0.99**	**0.99**	**−0.5 ± 2.3**
**Vol SA**	**1.80 ± 0.3**	**1.78 ± 0.3**	**0.04 ± 0.03**	**0.05**	**0.97**	**0.99**	**0.02 ± 0.04**

**R Leg**	**11.5 ± 2.5**	**10.4 ± 3.3**	**0.7 ± 0.7**	**1.1**	**0.80**	**0.88**	**0.1 ± 1.1** **
**Vol SA**	**0.36 ± 0.1**	**0.36 ± 0.04**	**0.02 ± 0.01**	**0.02**	**0.80**	**0.90**	**0.01 ± 0.02**

**L Leg**	11.7 ± 2.5	11.6 ± 2.1	0.9 ± 0.8	1.2	0.77	0.87	0.1 ± 1.2**
**Vol SA**	0.36 ± 0.1	0.36 ± 0.04	0.02 ± 0.02	0.02	0.75	0.87	0.01 ± 0.021

**Head**	6.1 ± 1.2	6.3 ± 1.0	0.5 ± 0.3	0.6	0.81	0.87	−0.2 ± 0.5**
**Vol SA**	0.19 ± 0.02	0.18 ± 0.02	0.01 ± 0.01	0.01	0.74	0.87	0.01 ± 0.012

**R Arm**	3.9 ± 1.2	3.6 ± 1.1	0.4 ± 0.4	0.5	0.87	0.89	0.3 ± 0.4*
**Vol SA**	0.18 ± 0.03	0.17 ± 0.03	0.01 ± 0.01	0.01	0.83	0.92	0.003 ± 0.01

**L Arm**	3.8 ± 1.1	3.7 ± 1.0	0.4 ± 0.3	0.5	0.84	0.90	0.2 ± 0.4
**Vol SA**	0.17 ± 0.03	0.17 ± 0.03	0.01 ± 0.01	0.01	0.79	0.89	−0.0005 ± 0.01

**Trunk**	41.9 ± 16.2	44.1 ± 18.2	3.1 ± 3.8	4.9	0.95	0.96	−2.2 ± 4.4**
**Vol SA**	0.61 ± 0.1	0.56 ± 0.1	0.1 ± 0.03	0.06	0.96	0.98	0.05 ± 0.032

*, p < 0.01 and

**, p < 0.001 for Bland-Altman slope

*** all p < 0.001.

Abbreviations: CCC, concordancecorrelation coefficient; GT, ground-truth; L, left; MAE, mean absolute error; Pred, predicted; R, right;Ref, reference; RMSE, root-mean square error.

## Data Availability

Data described in this manuscript will be made available upon request and approval by the investigators.

## Abbreviations

BIA: bioimpedance analysis
CCC: concordance correlation coefficient
DXA: dual-energy X-ray absorptiometry
MAE: mean absolute error
PC: principal component
RMSE: root mean square error
